# Identifying protein complexes in PPI network using non-cooperative sequential game

**DOI:** 10.1038/s41598-017-08760-x

**Published:** 2017-08-21

**Authors:** Ujjwal Maulik, Srinka Basu, Sumanta Ray

**Affiliations:** 1 Department of Computer Science and Engineering, Jadavpur University, Kolkata, 700108 India; 2 Department of Engineering and Technological Studies, University of Kalyani, Kalyani, West Bengal India; 3 Department of Computer Science and Engineering, Aliah University, Kolkata, 700156 India

## Abstract

Identifying protein complexes from protein-protein interaction (PPI) network is an important and challenging task in computational biology as it helps in better understanding of cellular mechanisms in various organisms. In this paper we propose a noncooperative sequential game based model for protein complex detection from PPI network. The key hypothesis is that protein complex formation is driven by mechanism that eventually optimizes the number of interactions within the complex leading to dense subgraph. The hypothesis is drawn from the observed network property named small world. The proposed multi-player game model translates the hypothesis into the game strategies. The Nash equilibrium of the game corresponds to a network partition where each protein either belong to a complex or form a singleton cluster. We further propose an algorithm to find the Nash equilibrium of the sequential game. The exhaustive experiment on synthetic benchmark and real life yeast networks evaluates the structural as well as biological significance of the network partitions.

## Introduction

Many cellular activities are carried out by proteins which physically interact with each other to form stoichiometrically stable complexes. These complexes interact with individual proteins or other complexes to form functional modules. The functional modules are observed to work in a coherent fashion even in the relatively simple model organisms like Saccharomyces cerevisiae^1^. Study of the entire set of complexes from the data describing the physical interactions among proteins, is thus essential to understand the complex formations, and the higher level cellular organization^2^.

New technological advancement in biotechnology have resulted in large-scale physical protein-protein interaction (PPI) data for different organisms which is used to reconstruct the PPI network. The PPI network can be described as an undirected graph where a node represents a protein and an edge represents the interaction between two proteins. Protein complexes constitute modular units within the PPI networks^3^. From a biological perspective, this modularity is a division resulted of evolution to provide robustness against mutation and chemical attacks^4^. From a topological perspective, this modularity is a result of proteins within complexes being densely connected to each other than to the rest of the network^1, 5, 6^. Thus the computational approaches to protein complex identification from PPI data can be formulated as dense subgraph detection from PPI networks.

Since last decade several computational methods have been developed to infer the protein complexes where a protein complex is defined as a dense subgraph. A large class of existing literature for protein complex identification is based on network motif identification like clique finding approaches in refs 3, 7–9. The motif identification based methods fail to identify the protein complexes of irregular shapes. Another class of graph clustering techniques based on global criteria^10^ suffers from issues like resolution limit. Local search based approaches, employing merging or growing of clusters have proved to be more efficient. A popular local search based method, Molecular Complex Detection (MCODE) algorithm, proposed in ref. 11, starts by assigning certain weights to all the nodes based on their the local neighborhood density. The method then iteratively adds vertices having weights above certain threshold, starting with the maximum weighted node as a seed node. Restricted Neighborhood Search Clustering (RNSC) proposed in ref. 12, uses a cost-based local search algorithm to minimize the number of intra-cluster and inter-cluster edges. The method starts from an initial random solution, and iteratively moves on to form groups by assigning each vertices to a group which reduces the general cost. Recently, an overlapping neighborhood expansion mechanism based approach, ClusterONE, for detecting overlapping protein complexes is proposed in ref. 2. ClusterONE uses a greedy search procedure to find highly cohesive groups of vertices. The search process is performed repeatedly considering different seeds to form multiple, possibly overlapping groups. To obtain the resulting complexes, the overlapping groups are merged based on their overlap scores.

Other popular and recent developed methods for protein complex identification includes Affinity Propagation^13^, multi-objective based framework^14, 15^, PPSampler2^16^ and PEWCC^17^. Affinity Propagation^13^ groups data points based on their similarity. All data points are initially considered as potential “exemplars”. The algorithm first finds sub-paths that allow easy message exchanges between nodes. In subsequent steps, message exchange between the nodes are continued until a high quality cluster is formed. The multi-objective based framework^14^ optimizes two objective functions, one is based on density of a module and another on the Gene Ontology based semantic similarity among proteins, resulting in dense and functionally homogeneous protein complexes. PPSampler2^16^ is a modification of its previous version PPSampler^18^ which partitions proteins into clusters based on a scoring function generated from the topological properties of the PPI network. A novel algorithm called PEWCC is proposed in Zaki *et al*., where reliability of the protein interaction data is assessed before partitioning the interaction network. PEWCC performs weighted clustering to partition the refined interaction networks into maximal cliques which serve as protein complexes.

A common limitation of the above mentioned approaches is that they ignore the self-organizing nature of the protein complexes, primarily triggered by the biochemical factors including hydrophilic energy, electrostatic forces between the proteins. Game theory, a natural choice of framework to model the self-organizing nature, could focus on the underlying factors that drive the protein complex formation. Bohl *et al*.^19^, reviews game theoretical concepts in cell biology and molecular biology focusing on the subcellular level by considering viruses, genes, and molecules as players. The existing works mostly uses two player simultaneous game for protein complex identification.

In this paper, we model the problem of protein complex detection as a multi-player sequential move non-cooperative game that models the proteins as rational players. The underlying hypothesis for the model are: (i) proteins always act in selfish manner: even when the proteins forms complexes it only optimizes its own objective(s) (ii) protein complexes are dense substructures of low diameters: most of the real life networks tend to demonstrate small world property, and (iii) there might exist some proteins which do not belong to any complex: this could be due to the physio-chemical properties. We formulate a Partial Dense vertex Cover (PDC) game for protein complex identification. The strategy set of a player is determined by a preference relation which is a transitive, reflexive function over the set of all possible vertex covers. The Nash equilibrium of the game is the minimum partial dense vertex cover. We propose an algorithm, NashPDC, to find the Nash equilibrium of the PDC game. The resulting partition generated by NashPDC represents the protein complexes.

A thorough experiment is carried out by comparing the performance of the proposed method with that of the other well-known methods such as clusterONE^2^, MCODE^11^, RNSC^12^, MCL^20^, PPSampler2^16^ and PEWCC^17^. The biological relevance of the identified complexes are assessed by gene ontology and pathway based analysis. The experiment uses synthetic benchmark network and real life yeast PPI network for evaluation. The experimental results show a significant improvement achieved by the proposed approach over the other methods.

## Materials and Methods

We represent a PPI network using an undirected graph *G* = (*V*, *E*) where a node *v* ∈ *V* represents a protein and an edge *e* ∈ *E* represents the interaction between two proteins. In this section, we discuss the proposed method which is based on the following basic concepts of graph theory and non-cooperative game theory.

Given an unweighted graph *G* = (*V*, *E*), *G*(*S*) denote the subgraph induced by *S* on *G* where *S* ⊆ *V*. The set of edges of *G*(*S*) is denoted by *E*(*G*(*S*)). For any vertex *v* ∈ *V*, the *p* ^*th*^ order open neighborhood, *N*_*p*_(*v*), is the set of vertices connected to *v* by a path of length less than or equal to *p*. The closed *p* ^*th*^ order neighborhood of vertex *v* is *N*_*p*_[*v*] = *N*_*p*_(*v*) ∪ {*v*}. The degree of vertex *v* ∈ *V*, *d*_*v*_ = |*N*_1_(*v*)|. The degree density, *α*(*G*), is defined as the ratio of the minimum degree of *G* to the maximum possible degree, i.e. $\alpha (G)=\frac{mi{n}_{v\in V}{d}_{v}}{|V|-1}$. The edge density, *δ*(*G*), is defined as the ratio of the number of edges in *G* to the total number of possible edges, i.e. $$\delta (G)=\frac{|E(G)|}{(\begin{array}{c}|V|\\ 2\end{array})}$$. The radius, *R*(*G*), also termed as the minimum eccentricity of *G* is defined as the maximum shortest path between any two pairs of nodes, i.e. *R*(*G*) = *max*_*u*,*v*∈*V*_|*SP*(*u*, *v*)| where *SP*(*u*, *v*) denotes the shortest path between *u*, *v*. The local transitivity of a vertex *v*, denoted by *t*(*v*) is defined as the ratio of the triangles connected to the vertex and the triples centered on the vertex, i.e. $t(v)=\frac{|\{(u,v),(v,w),(u,w)|(u,v),(v,w),(u,w)\in E(G)\}}{|\{(u,v),(v,w)|(u,v),(v,w)\in E(G)\}|}$. Further, the term cover indicate a set of vertices and a graph partition is defined as a set {*C*_1_, *C*_2_, …, *C*_*K*_} (*K* is a positive integer and *K* ≤ |*V*|) such that – (i) ∀ *k* ∈ {1, …, *K*}, *C*_*k*_ ≠ ∅, (ii) ${\cup }_{k=1}^{K}{C}_{k}=V$ and (iii) ∀ *k*, *l* ∈ {1, …, *K*} with *k* ≠ *l*, *C*_*k*_ ∩ *C*_*l*_ = ∅.

In non-cooperative game theory, strategic form game models the interaction between a finite set of *N* rational players. In strategic form game a player’s decision problem is to choose a strategy that will counter best the strategies adopted by the other players. Each player is faced with this problem and the players can be thought of as simultaneously choosing their strategies from the respective strategy sets. A strategic form game is modeled by a three tuple $(N,({S}_{i}{)}_{i\in N},({\succcurlyeq }_{i}{)}_{i\in N})$ where -

- *N* is a finite set of rational players
- *S*_*i*_ denote the strategies or actions of player *i* while *S* = x_*i*∈*N*_ *S*_*i*_ is called the set of action profiles (or strategy profiles)
- ${\succcurlyeq }_{i}$ denote the preference relation which is a reflexive $(a\,\succcurlyeq \,a)$, transitive $(a\,\succcurlyeq \,b\,{\rm{and}}\,b\,\succcurlyeq \,c,\,{\rm{implies}}\,a\,\succcurlyeq \,c)$, total (for all elements *a*, *b* either $a\,\succcurlyeq \,b$ or $b\,\succcurlyeq \,a$) binary relation on the set of action profiles. We write $a\,\succ \,b$ if $a\,\succcurlyeq \,b$ but not $b\,\succcurlyeq \,a$. Intuitively, $a\,\succ \,b$ means that strategy *b* is preferable to *a*. The preference relation may also be defined based on the outcomes of the strategies.

It is assumed that in the strategic form game the set of strategies and the players’ preference relations are known to all the players. The only uncertainty concerns the actions chosen by the players. For *i* ∈ *N* let *s*_−*i*_ ∈ *S*_−*i*_ where *S*_−*i*_ denote the action profile of all the players in *N*\*i*. The best responses of player *i* given the actions of other players, *s*_−*i*_, is defined as

$${B}_{i}({s}_{-i})=\{{s}_{i}\in {S}_{i}|({s}_{-i},{s}_{i})\,{\succcurlyeq }_{i}\,({s}_{-i},{s}_{i}^{^{\prime} }),\forall {s}_{i}^{^{\prime} }\in {S}_{i}\}$$

An action profile ${s}^{\ast }=({s}_{1}^{\ast },{s}_{2}^{\ast },\ldots ,{s}_{n}^{\ast })$ such that for each $i\in N,{s}_{i}^{\ast }\in {B}_{i}({s}_{-i})$ is in Nash equilibrium. In other words, in Nash equilibrium no player *i* has a profitable deviation from *s* ^*^.

### The framework

The proposed framework is based on the concept of partial dense vertex cover, defined as below.

**Definition 1**. *Given an unweighted graph G* = (*V, E*), *a partial dense vertex cover C*(*G*) *is a collection of subsets of V*, {*C*_1_, … *C*_*k*_} *with the properties*:

1. ∀*i* ∈ {1, … k}, ∅ ≠ C_i_ ⊆ V
2. ∀*i*, j ∈ {1, …, k} with i ≠ j, C_i_ ∩ C_j_ = ∅
3. *C*_1_ ∪ … ∪ *C*_*k*_ ⊆ *V*
4. ∀*i* ∈ {1, … *k*}, *R*(*G*(*C*_*i*_)) ≤ *p*
5. ∀*i* ∈ {1, … *k*}, *α*(*G*(*C*_*i*_)) ≥ *λ and δ*(*G*(*C*_*i*_)) ≥ *γ*
6. ∀*i* ∈ {1, … *k*}, *C*_*i*_ *is a locally maximal cover*.
7. *the residual graph* $G(V\backslash {\cup }_{i=1}^{k}{C}_{i})$ *does not contain any induced subgraph satisfying the other conditions*.

*Here* 0 < λ ≤ γ ≤ 1 *and p is a positive integer*.

A minimum partial dense vertex cover is a partial dense vertex cover with the minimum value of *k*. In this paper, the terms cover and coalition have been used interchangeably.

The proposed method models the protein-complex detection method as a partial dense vertex cover (PDC) strategic form game, $PDC=(N,{({S}_{i})}_{i\in N},{({\succ }_{i})}_{i\in N})$ as discussed below.

- Each player *i* from the set of rational players *N* represents a node *i* ∈ *V* in *G*.
- The strategy set *S*_*i*_ of a player *i* ∈ *N* is to – (i) propose a coalition, by sending out joining requests (ii) accept a joining request (iii) reject a joining request and (iv) leave a coalition. Once a player accepts and joins a coalition he is not allowed to propose a new coalition. A player if rejects a coalition joining request or leaves a coalition can subsequently propose a new coalition. A proposer, if do not receive satisfactory response may choose to leave the coalition.
- ${\succ }_{i}$ is the preference relation of a player *i* over the set of possible partitions. The preference relation determines the quality of the partial dense vertex cover. A player prefers to belong to a minimum partial dense vertex cover or to stay alone than to belong to any random partition.

Given the preference relation ${\succ }_{i}$, the Nash equilibrium of the PDC is a partition where no player can gain from unilateral deviation. For a PDC game, a partition *p* ^*^ is in Nash equilibrium, if for all other partitions *p* and for every player *i*, ${p}^{\ast }{\succcurlyeq }_{i}p$ when all the players in *N*\*i* plays their best response strategy. In other words, a minimum partial dense vertex cover is a Nash equilibrium of PDC game and vice versa. There may exist multiple Nash equilibriums. We eliminate the two trivial partitions i) where a single cover or a grand coalition forms and ii) where all the players form singleton coalitions.

The following example illustrates the above framework.

**Figure 1:**
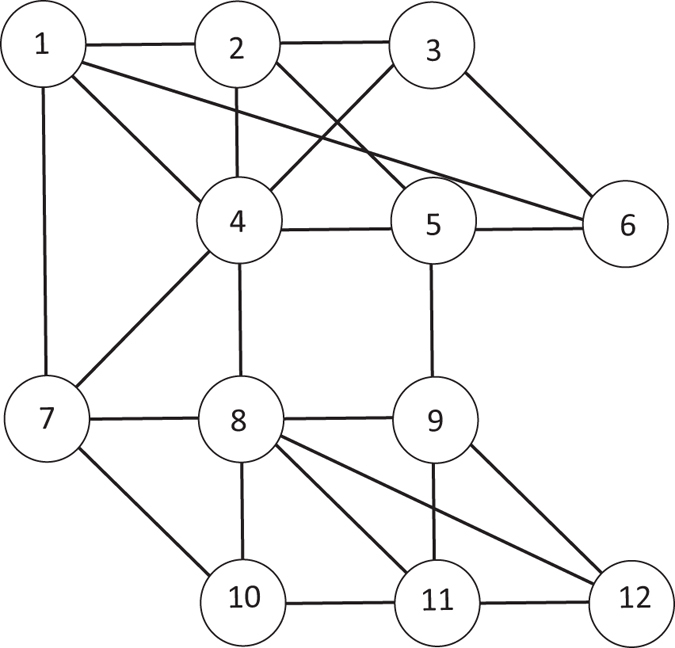
Toy example of the framework. The minimum PDC {{1, 2, 3, 4, 5, 6}, {7, 8, 9, 10, 11, 12}} is in Nash equilibrium as no player can gain from unilateral deviation.

**Example 1**. Figure 1 *shows a graph G with* 12 *nodes. We induce the PDC game on the graph G so that every node of G is mapped to a player in PDC*. *For λ* = 0.6, *γ* = 0.65 *and p* = 2 *the preference relation of the players are given as below*.

$$\begin{array}{c}\{1,2,3,4,5,6\}{\succ }_{3}\{1,2,3,4,5\}{\succapprox}_{3}\ldots \\ \{1,2,3,4,5,6\}{\succ }_{4}\{1,2,3,4,5\}{\succ }_{4}\{1,2,3,4\}{\succ }_{4}\ldots \\ \{1,2,3,4,5,6\}{\succ }_{5}\{1,2,3,4,5\}{\succ }_{5}\{1,2,3,5\}{\succ }_{5}\ldots \end{array}$$

*The partition* {{1, 2, 3, 4, 5, 6}, {7, 8, 9, 10, 11, 12}} *is in Nash equilibrium, as no player can gain from unilateral deviation*.

We propose an algorithm, Nash equilibrium based Partial Dense vertex Cover Detection (NashPDC) to find the Nash equilibrium of the game PDC. The basic working principal of the Algorithm 1 is described below.

- The players are ranked based on their geometric mean of degree and local transitivity (in descending order). This forms the rule of order. The first player in the rule of order is the one with the maximum links and maximum local transitivity. This is one of the ways to prioritize proteins. However, other approaches may be used.
- The first player *i* of rule of order proposes a coalition *C* which is the *p* ^*th*^ order bounded neighborhood of *i*.
- Each player *j* in *C* accepts the coalition joining request only if that is his best response strategy, otherwise rejects the invitation. Once all the players either accept or reject the coalition joining request, a stable coalition *C* forms. Subsequently, all the players in *C* quit the game.
- The game play is continued by repeating the above steps with the players who do not belong to any coalition until no more stable coalition can be formed.

**Algorithm 1:**
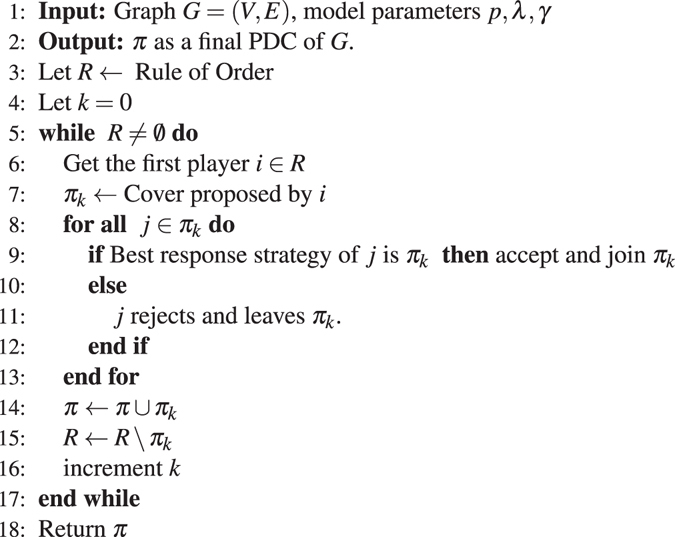
NashPDC.

### Complexity

At the worst case, every node sends a coalition request. Selecting a player from the rule of order takes *O*(*logN*) time. For each proposed coalition at most ${d}_{max}^{p}$ number of nodes are processed where *d*_*max*_ is the maximum degree in *G*. Thus the worst case complexity of the algorithm 1 is $O(N(\mathrm{log}\,N+{d}_{max}^{p}))$. With *p* = 2 the complexity reduces to $O(N(\mathrm{log}\,N+{d}_{max}^{2}))$.

## Results

In this section, we evaluate the performance of our proposed method with that of some state-of-the art techniques. The evaluation is conducted on both synthetic benchmark and real life yeast PPI network data sets.

### Dataset description

To test the correctness of the proposed method we first use the synthetic benchmark networks generated using the popular Girvan Newman (GN) model^21^. GN benchmark is a special case of the planted 4-partition model where a graph with 128 vertices are partitioned into 4 groups (modules) with 32 vertices each. The inter-module edges of GN model is controlled by the mixing parameter *μ*. In other words, the mixing parameter *μ* controls how well the modules are defined by controlling the number of edges with which a module is connected to the rest of the graph. When the value of *μ* is strictly less than 0.5, the expected number of links joining a node to others in different groups is less than those in the same group. This yields well defined groups (modules). As the value of *μ* drops to 0.5 or below, it becomes difficult to identify a module boundary from the rest of the network. Irrespective of the size and sparsity of the networks, GN benchmark dataset is a popular choice to test the correctness of different module detection algorithms and compare their performances. By varying the value of *μ* we generate networks with well defined modules as well as the networks with modules that are not so clearly distinguishable from the rest of the network. The later is a case similar to real life scenarios where all modules are not necessarily well defined. The code to generate the GN networks are freely available at the site https://sites.google.com/site/santofortunato/inthepress2.

Further we use real life yeast PPI networks - two experimental yeast PPI datasets (Gavin *et al*.^22^, Krogan *et al*.^23^), yeast PPI interaction derived from DIP^24^ and a manually curated high-quality yeast dataset from MIPS^25^. The Krogan dataset has two variants, namely, the core data set which is referred to as Krogan core and the extended data set which is referred to as Krogan extended. The key topological properties of the PPI networks built from these dataset are given in Table 1. For comparing the resulting protein complexes with benchmark we downloaded gold standard data from the site http://yeast-complexes.russelllab.org/. It consists of 491 experimentally verified yeast protein complexes. We consider this as benchmark and compute the extent of overlap with the resulting clusters.

**Table 1:** The topological parameters of the PPI datasets. *N* is the number of nodes, *E* is the total edge count, *δ* the network density defined as $$E/(\begin{array}{c}N\\ 2\end{array})$$, *d*_*avg*_ is average degree and *CC* is the clustering coefficient. Krog_Cr and Krog_Ex are the Krogan Core and Krogan Extended data sets respectively.

Dataset	*N*	*E*	*δ*	*davg*	*CC*
DIP	4667	21619	0.002	9.203	0.049
MIPS	3950	11119	0.001	5.529	0.093
Gavin	1465	7672	0.007	10.474	0.531
Krog_Cr	2708	7122	0.002	5.260	0.188
Krog_Ex	3674	14342	0.002	7.807	0.120

### Validation on synthetic benchmark

We first test the performance of the proposed method on synthetic benchmark networks generated using GN model. The inter-module edges of GN model is controlled by the mixing parameter *μ*. We consider *μ* = {0.1, 0.2, …, 0.7}. To simulate the real life modules which are not very clearly distinguishable from the rest of the network, we vary the value of *μ*. To avoid any bias in the results obtained by conducting the experiments on a single GN synthetic network, we generate 100 instances of every configuration of GN model. The results obtained for each network configuration are then averaged and reported.

**Figure 2:**
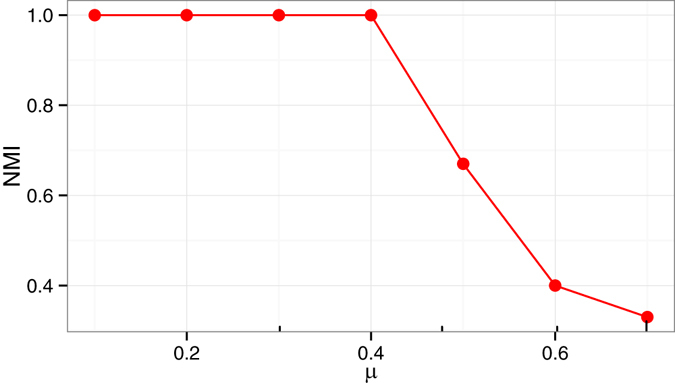
The figure shows the NMI values obtained by running the proposed method on GN network instances with different values of *μ*.

To quantitatively measure the correspondence between the ground-truth modules and the identified modules we use the metric Normalized Mutual Information (NMI). NMI, defined as below, measures the similarity between two partitions based on entropy^26, 27^. For two given partitions *π* ^*a*^,*π* ^*b*^, NMI is:

$$\frac{-2{\sum }_{i=1}^{{k}_{a}}{\sum }_{j=1}^{{k}_{b}}{n}_{ij}^{ab}(\mathrm{log}\,\frac{{n}_{ij}^{ab}.n}{{n}_{i}^{a}.{n}_{j}^{b}})}{{\sum }_{i=1}^{{k}_{a}}{n}_{i}^{a}(\mathrm{log}\,\frac{{n}_{i}^{a}}{n})+{\sum }_{j=1}^{{k}_{b}}{n}_{j}^{b}(\mathrm{log}\,\frac{{n}_{j}^{b}}{n})}$$

where ${n}_{i}^{a}$ is the number of nodes in cluster ${C}_{i}^{a}\in {\pi }^{a}$ and ${n}_{ij}^{ab}$ is the number of common nodes between cluster ${C}_{i}^{a}\in {\pi }^{a}$ and ${C}_{i}^{b}\in {\pi }^{b}$. The value of NMI ranges between 0 to 1. Higher the value, the more similar the two partitions are with the maximum value 1 indicating two identical partitions. Figure 2 shows the NMI values for different values of *μ*. As shown in the figure, the proposed method correctly identifies all the modules for the values of *μ* upto 0.4. For higher values of *μ* as the inter module edge count becomes equal or more than the intra module edge count, identifying a module, thus, becomes difficult. As a result the NMI falls sharply when *μ* ≥ 0.5. The experiment shows the accuracy of the proposed method in identifying well defined modules.

### Validation on null model

To verify the performance of the algorithm on the null model, we test our performance on random Erdös-Réyni (E-R) graphs^28^. The nodes being randomly connectedly, the E-R network do not exhibit any modular structure. A good dense cluster detection algorithm should not identify any significant structure from E-R network. We run the proposed method on the E-R graphs. The proposed method do not identify any significant module. The experiment shows that the proposed method works correctly on null model.

### Validation on noisy synthetic data

Presence of noise is a very common phenomenon when dealing with complex real life data sets. Noisy data occur due to many reasons including but not limited to erroneous measurements, sampling bias. The study of stability of a method against noise is thus essential. We study the stability of the proposed method when the endpoints of the edges of a graph is rewired to a random vertex with given rewiring probability (*ρ*). The initial graphs are generated using GN model. We measure the performance by computing the NMI between the known partition of the initial graph and the partition obtained from the perturbed graph for various values of *ρ*.

**Figure 3:**
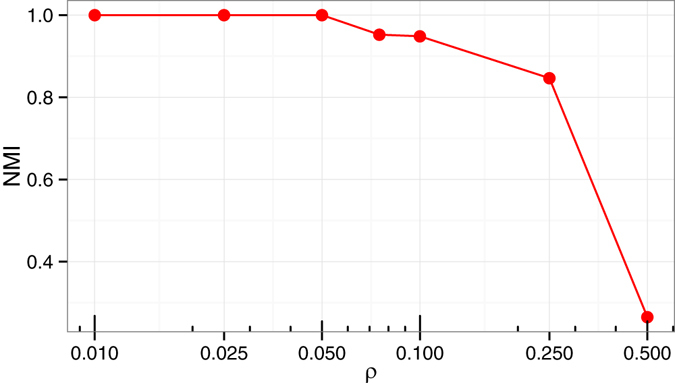
The figure shows the NMI values obtained by running the proposed method on the perturbed GN network instances with different values of edge rewiring probability (*ρ*).

Figure 3 shows the NMI values for different values of *ρ*. As shown in the figure, the proposed method correctly identifies all the modules for the values of *ρ* upto 0.05. When *ρ* is above 0.25 the value of NMI drops sharply. The experiment shows that the proposed method correctly identifies the modules when a certain level of random noise present in the data and is thus robust against random noise.

### Validation using connectivity density

To validate the identified modules from topological perspective, we use the connectivity density measure^29^. The connectivity density of a module *M* is defined as follows:

$$\frac{{\sum }_{{n}_{i}\in M}d({n}_{i})}{N},$$

where *d*(*n*_*i*_) represents degree of node *n*_*i*_ within the module *M*, *N* represents total number of connections. It simply denotes the ratio of total degrees of nodes within the module to the total number of connections. The experiment studies the relative change in the connectivity density when an identified protein complex is shifted a little. A shifted (replaced) module is obtained by randomly replacing a small portion of the proteins in a module with the same number of proteins outside of that module in such a way that the replacement proteins are connected with the proteins in the module but do not belong to it. For an identified protein module, a little shift is expected to decrease the connectivity density, which should not be expected from a random module. In addition, shift in random modules may results either increase or decrease in connectivity density. The experiment is carried out on the real life yeast PPI networks.

**Figure 4:**
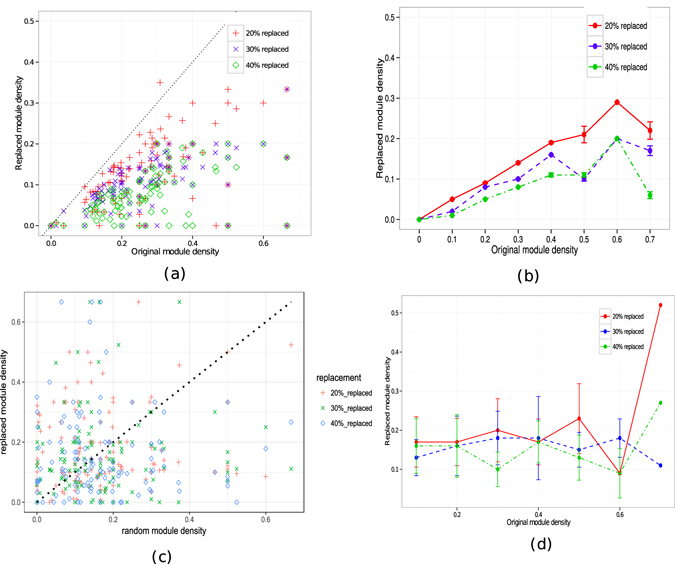
Comparing the density of original modules and newly constructed modules for Gavin dataset. (**a**) Each point in the scatter plot represents the density of an original module (x-axis) and newly constructed module (y-axis). The dashed line (y = x) indicates the points where the connectivity density is the same for the original module and replaced module. Any data point above the line corresponds to the case where the replaced modules have higher connectivity density, while data points below the line represent the case where the replaced module has lower connectivity density than the actual functional module. (**b**) Shows the summary statistics - the vertical axis shows the density of the original modules and the horizontal axis shows the average density of the replaced modules with standard deviation. Panel (c,d) shows the same for randomly constructed modules.

Figure 4(a,b) shows the results averaged over 300 randomization experiments. Figure 4(a) is a scatter plot of the densities of identified modules and replaced modules. As shown in Fig. 4(a) for most of the modules, 20% component replacement causes the density to decrease by a significant amount dropping below the original density. With the higher rate of replacements (30% and 40%), the density decrease is even higher. Figure 4(b) shows the summary statistics of the changes in the replaced module density. As shown in the figure, on an average the replaced module density is nearly 50% of the original module density for 20% component replacement and even lower for higher replacements. The observation suggests that the identified modules are indeed densely connected local subgraphs, and thus are good candidates for functional modules in the yeast protein network.

To know whether the obtained results is trivial and can be easily expected for any random modules we have performed an analysis. For this, we have repeated the same experiment for randomly selected modules from the PPI network. Figure 4(c,d) shows the scatter plots of the densities of random as well as replaced modules. It is observed that there is no distinct pattern which distinguishes the densities of modules with 20, 30 and 40% replacement.

### Evaluating structural quality using ground truth

In this experiment the performance of the proposed method is compared with the base line methods by using metrics that measure the correspondence between the predicted complexes and the reference complexes. The metrics used for evaluation are: (i) Jaccard index based performance metric, matching score(*D*), (ii) sensitivity(*Sn*), positive predictive value(*PPV*), and accuracy(*Acc*)^2, 30^.

Let *P* = {*P*_1_, *P*_2_, … *P*_*n*_} and *R* = {*R*_1_, *R*_2_, … *R*_*m*_} are the sets of predicted complexes and reference complexes, respectively. The matching score is the geometric mean of *S* and *T*, defined as:

1 $$D=\sqrt{S\times T}.$$

where *S* is the average predicted complex-wise overlap defined as $S=\frac{1}{n}{\sum }_{i=1}^{n}O{v}_{i}$, *T* is the average reference complex-wise overlap defined as $T=\frac{1}{m}{\sum }_{j=1}^{m}O{v}_{j}$, *Ov*_*i*_ is the predicted complex-wise overlap defined as $O{v}_{i}=ma{x}_{j=1}^{m}{o}_{ij}$, *Ov*_*j*_ is the reference complex-wise overlap defined as $O{v}_{j}=ma{x}_{i=1}^{n}{o}_{ij}$, *o*_*ij*_ is the overlap score defined as ${o}_{ij}=\frac{|i\cap j|}{|i\cup j|}$, *i* ∈ *P* and *j* ∈ *R*. Higher the value of *D* indicates better involvement of predicted complexes to the reference complexes and vice-versa.

The metrics *Sn*, *PPV*, *Acc* are defined on a contingency table *T*, where every element *t*_*ji*_ indicates the number of common proteins between reference complex *j* and predicted complex *i*. The General Sensitivity (*Sn*) of a clustering result is defined as:

2 $$Sn=\frac{{\sum }_{j=1}^{m}{N}_{j}S{n}_{j}}{{\sum }_{j=1}^{m}{N}_{j}}$$

where *N*_*j*_ is the number of proteins belonging to complex *j* and *Sn*_*j*_ is the reference complex-wise sensitivity for reference complex *j* defined as $S{n}_{j}={\max }_{i=1}^{n}\frac{{t}_{ji}}{{N}_{j}}$.

The General PPV(*PPV*) of a clustering result is defined as:

3 $$PPV=\frac{{{\sum }_{i=1}^{n}{T}_{.i}PPV}_{i}}{{\sum }_{i=1}^{n}{T}_{.i}}$$

where ${T}_{\mathrm{.}i}={\sum }_{j=1}^{m}{t}_{ji}$ is the marginal sum of a column *i* in the contingency table *T* and *PPV*_*i*_ is the predicted complex-wise positive predictive value for predicted complex *i* defined as $PP{V}_{i}={\max }_{j=1}^{m}\frac{{t}_{ji}}{{T}_{.i}}$.

Since *Sn* is maximum when every protein is assigned to the same cluster, while the *PPV* is maximum when every protein is in its own cluster, it is necessary to balance the two measures. The Geometric Accuracy (*Acc*) represents a trade-off between sensitivity and the positive predictive value and is defined as:

4 $$Acc=\sqrt{{S}_{n}\times PPV}\mathrm{.}$$

The advantage of taking the geometric mean is that it yields a low score when either the *Sn* or the *PPV* metric is low. High accuracy value thus indicates a high performance in terms of both *Sn* and *PPV*.

The results of evaluation are shown in Tables 2 and 3. In Table 2 the performance of the proposed method is compared with other five baseline methods, viz., MCODE, MCL, ClusterONE, RNSC, PPSampler2 and PEWCC in terms of the matching score *D*. As shown in the table, unlike other methods, the proposed method performs consistently well on all the data sets. The proposed method outperforms MCODE and MCL in most of the data sets. ClusterONE method attains higher *D* value for DIP, GAVIN and Krogan_Cr data sets while the same attains lower *D* value for other data sets. On the contrary, RNSC method attains higher *D* value for MIPS and Krogan_Ex data sets while the same attains lower *D* value for other data sets.

**Table 2:** Comparisons of performance of different algorithms using matching score.

Method	Matching score					#predicted complex				
	DIP	MIPS	Gavin	Krog_Cr	Krog_Ex	DIP	MIPS	Gavin	Krog_Cr	Krog_Ex
MCODE	0.2738	0.0795	0.2481	0.1109	0.0890	122	98	104	43	78
MCL	0.2930	0.0769	0.2628	0.1208	0.1290	340	244	155	110	178
ClusterONE	0.3024	0.0901	0.3384	0.1281	0.1187	258	158	189	240	144
RNSC	0.2890	0.1001	0.2533	0.1078	0.1381	102	54	68	88	35
PPSampler2	0.2728	0.0829	0.2674	0.1102	0.1348	254	130	499	261	155
PEWCC	0.2876	0.0938	0.2355	0.1032	0.1214	96	63	84	47	48
Proposed	0.2938	0.0917	0.2711	0.1134	0.1373	124	59	74	119	41

Krog_Cr and Krog_Ex are the Krogan_Core and Krogan_Extended data sets respectively. Third column represents number of predicted complexes in each algorithm

**Table 3:** Comparisons of results with respect to sensitivity, specificity and accuracy.

Method	General Sensitivity					General PPV					Accuracy				
	DIP	MIPS	Gavin	Krog_Cr	Krog_Ex	DIP	MIPS	Gavin	Krog_Cr	Krog_Ex	DIP	MIPS	Gavin	Krog_Cr	Krog_Ex
MCODE	0.1168	0.0742	0.2807	0.1001	0.1291	0.4922	0.4709	0.5424	0.2568	0.3172	0.2397	0.1971	0.3902	0.1605	0.2024
MCL	0.2605	0.1588	0.3520	0.1342	0.1738	0.4464	0.4135	0.4231	0.2892	0.2130	0.3486	0.2563	0.3859	0.1970	0.1921
clusterONE	0.2135	0.0999	0.3731	0.1171	0.1534	0.4078	0.3890	0.4316	0.3210	0.3075	0.2951	0.1971	0.4013	0.1939	0.2172
RNSC	0.2901	0.1922	0.4021	0.1139	0.2135	0.6608	0.6048	0.3502	0.2901	0.3010	0.4348	0.3409	0.3753	0.1818	0.2535
PPSampler2	0.1786	0.0976	0.2408	0.1165	0.1786	0.6321	0.6029	0.3567	0.3189	0.3033	0.3360	0.2426	0.2931	0.1927	0.2327
PEWCC	0.2134	0.1126	0.2265	0.1349	0.1987	0.6676	0.6081	0.3245	0.2876	0.2774	0.3774	0.2617	0.2711	0.1970	0.2348
Proposed	0.2672	0.1233	0.4744	0.1211	0.2034	0.6831	0.6138	0.3620	0.3123	0.2787	0.4272	0.2751	0.4144	0.1945	0.2381

Table 3 shows the performance of the methods in terms of the metrics *Sn*, *PPV*, *Acc*. It can be noticed from the table that the proposed method performs consistently well in each of the datasets. In terms of *Sn* the proposed method outperforms MCODE and clusterONE in all the data sets. However, RNSC and MCL exhibit higher accuracy (*Acc*) than the proposed method on MIPS and KROGAN-extended data sets. It is also important to note that only the proposed method consistently gives good *Sn* value on all the data sets. As shown in the result no other method attain consistently high *Sn* on all the data sets.

**Figure 5:**
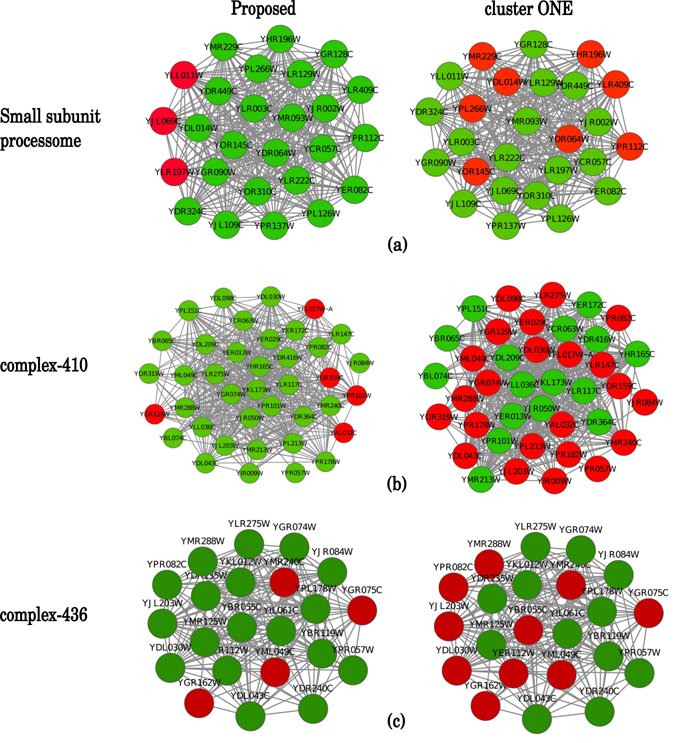
Figure shows visualization of three benchmark complexes: ‘Small subunit processom’ (panel-a),‘complex-435’ (panel-b) and ‘complex-410’ (panel-c) as detected by the proposed method and clusterONE. In the benchmark complexes, green nodes are predicted by the methods, while red nodes are not detected.

We further analyze the the complexes identified by the proposed method on Gavin dataset, and compare the same with that identified by clusterONE method as clusterONE attains second highest accuracy score after the proposed method. Among the experimentally verified benchmark complexes in Gavin data set, 10.62% complexes (52 complexes out of 490 complexes) are captured with more than 80% coverage by the proposed method. On the contrary clusterONE covers 8.16% benchmark complexes (40 out of 490) with more than 80% coverage. Figure 5 shows the layout of the three benchmark complexes: ‘Small subunit processom’, ‘complex-435’ and ‘complex-410’. Green and red nodes represent captured and non-captured nodes, respectively, by a particular method. From Fig. 5(a) it can be noticed that, for ‘Small subunit processom’ complex, the proposed method covers 89.66% proteins, while for clusterONE the percentage of coverage is 68.97%. Similarly, for the other two complexes, the coverage attained by the proposed method is much higher than that of clusterONE.

To further know the performance of the proposed method in large PPI data, we have utilized WI-PHI PPI database of yeast^31^, which consists 6,000 proteins and 50,000 PPIs. We run the proposed method on this data and compare the predicted clusters with the experimentally verified complexes. The results consists of 17 predicted clusters with minimum and maximum size eight and 39, respectively. The resulting sensitivity (*S*_*n*_ = 0.2384), positive predictive value (*PPV* = 0.4619) and accuracy (*Acc* = 0.3318) reveals that proposed method performed well in large PPI data.

**Figure 6:**
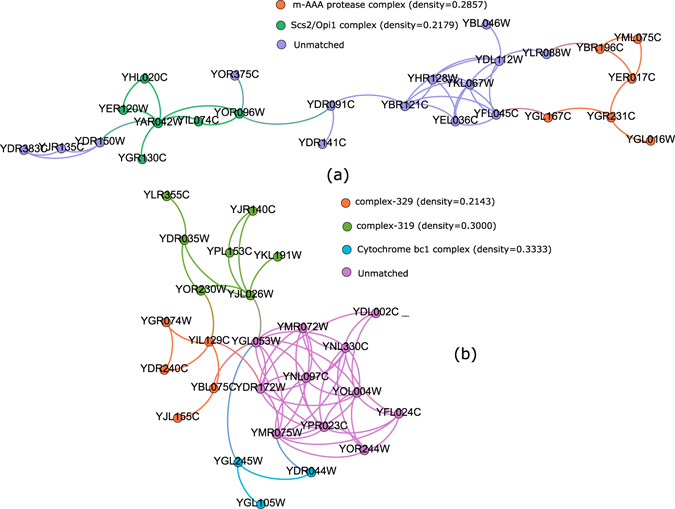
Figure shows two identified clusters (panel-a and panel-b) which match five benchmark complexes of low density:m-AAA protease complex, Scs2/Opi1 complex complex-329, complex-319 and Cytochrome bc1 complex.

To investigate whether the proposed method can detect sparse, low dense protein complexes we set the input parameter *α* (degree density) and *δ*(edge density) to lower values (*α* = 0.001, *delta* = 0.0008) and run the algorithm. We collected the resulting low dense clusters and compare these with benchmark protein complexes having lower density. Figure 6 shows two identified clusters which match with five benchmark low dense complexes such as: m-AAA protease complex, complex-329, Scs2/Opi1 complex, complex-319 and Cytochrome bc1 complex. It can also be noticed from the Fig. 6 that the unmatched proteins of the identified clusters exhibits high interactions among them and need to be explored further.

### Evaluating biological significance

#### Functional similarity of the identified complexes

It has been observed that proteins within a complex are functionally similar^14, 32, 33^. For understanding the biological roles and functions of proteins, functional similarity is a more informative measure compared to the structural and sequence similarity. The semantic similarity between Gene ontology (GO) terms is used to measure the functional similarity between proteins^34^. Here we use Relevance measure proposed in ref. 35 to compute functional similarity between the identified modules.

**Figure 7:**
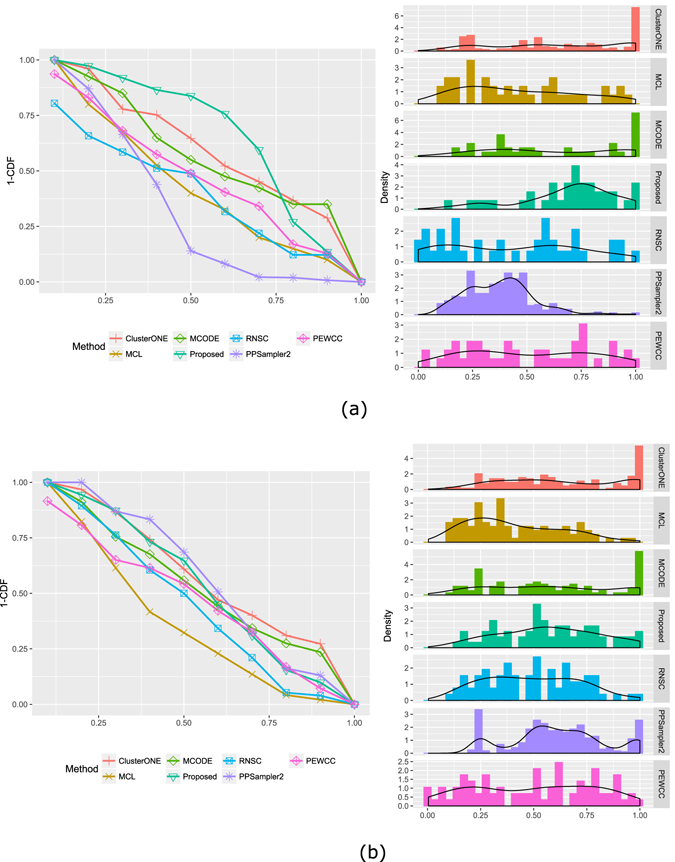
Distribution of functional similarity scores - the left pane show the fraction of identified modules having similarity score above certain value *x*, while the right pane show the distribution of similarity score. (**a**) Shows the results on Gavin data set and (**b**) shows the results on Krogan_Extended dataset.

Figure 7 shows the distribution of functional similarity scores for the identified modules. In Fig. 7(a) the left pane shows the fraction of identified modules having similarity score above certain value *x*, while the right pane shows the distribution of similarity score. As shown in the figure for Gavin data set more than 75% modules identified by the proposed method have semantic similarity score higher than 0.5 while for the MCODE, MCL, clusterONE and RNSC it is 53.66%, 39.02%, 61.34% and 48.78%. Similarly, as shown in Fig. 7(b) a large fraction of modules identified by the proposed method on the Krogan_Extended data set has similarity score above 0.5.

#### Gene ontology based analysis of the identified complexes

**Figure 8:**
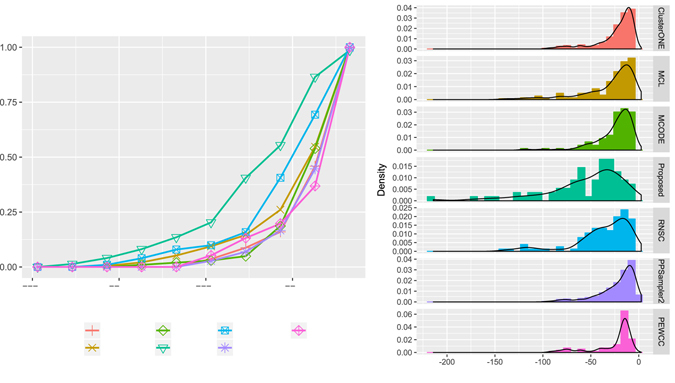
Distribution of p-values of the identified complexes on Gavin data set - (**a**) the left pane shows the fraction of identified modules having p-value below certain value *x*, (**b**) while the right pane shows the distribution of p-values.

We have performed a GO and pathway based study to biologically validate the identified protein complexes. GO represents an important resource to describe the functional characteristics of genes in a module. In Table 4 we provide the most significant GO terms, GO-id of biological process (BP) annotation of top 15 identified protein complexes in Gavin dataset. In Table 4 last column provides the p-value of each annotated GO-term. The p-value is computed by comparing the GO terms shared by the genes in the module to the background distribution of annotation. We have utilized a widely used webserver David functional annotation tool (https://david.ncifcrf.gov/) to perform the gene enrichment analysis. Here, the obtained p-values are calculated by using Fisher’s exact test. The obtained p-values are also subsequently adjusted for multiple comparisons using a Bonferroni correction based on the number of genes in the modules. The p-value of a gene module signifies the probability of observing at least *x* number of genes out of total *n* genes in the module annotated to a particular GO terms, given that the proportion of genes in the whole genome are annotated with that GO terms. So a p-value of a module closer to zero signifies that it is less likely to observe the annotation of a particular GO term to a group of genes occurs by chance. For comparison purpose we have computed the p-values of all the protein complexes identified by MCODE, MCL, clusterONE, RNSC and PPSampler2 algorithm. The distribution of p-values is given in Fig. 8. The left pane of the Fig. 8 shows the fraction of modules having p-value below certain value *x* while the right pane shows the distribution of p-values. As shown in the figure for any given p-value, the fraction of predicted complexes identified by the proposed technique is larger than the other methods.

**Table 4:** GO-terms, GO-id and p-value of top 15 identified complexes.

Sl No.	GO-id	GO-term	P-value
1	GO:0000375	RNA splicing, via transesterification reactions	8.60E-94
2	GO:0034660	ncRNA metabolic process	1.15E-76
3	GO:0000079	regulation of cyclin-dependent protein serine/threonine kinase activity	2.44E-68
4	GO:0009098	leucine biosynthetic process	3.39E-65
5	GO:0009101	glycoprotein biosynthetic process	3.06E-57
6	GO:0051603	proteolysis involved in cellular protein catabolic process	2.95E-56
7	GO:0042797	tRNA transcription from RNA polymerase III promoter	3.03E-54
8	GO:0010564	regulation of cell cycle process	2.44E-48
9	GO:0016192	vesicle-mediated transport	6.18E-48
10	GO:0006897	endocytosis	5.30E-45
11	GO:0022616	DNA strand elongation	1.03E-44
12	GO:0032543	mitochondrial translation	2.03E-39
13	GO:0006468	protein phosphorylation	4.17E-38
14	GO:0034965	intronic box C/D snoRNA processing	1.03E-37
15	GO:0006325	chromatin organization	1.88E-37

### Evaluation using aggregated ranking

**Figure 9:**
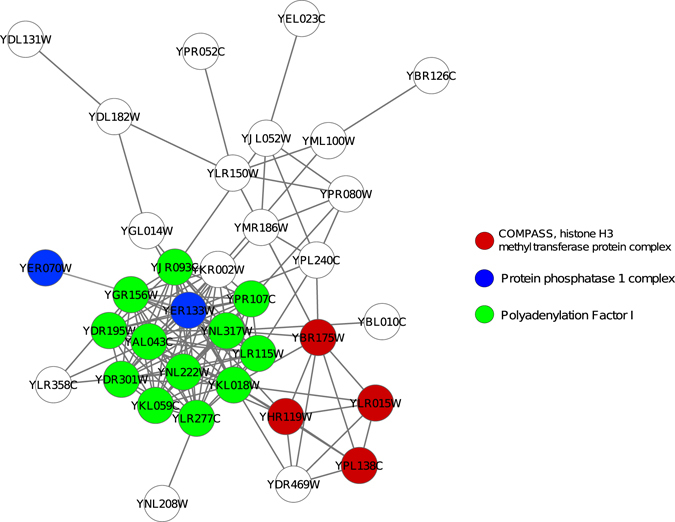
Top rank predicted complex, involved in three different benchmark complexes. Red, green and blue nodes are involved in the subunit of three benchmark complexes: COMPASS, histone H3 methyltransferase protein complex, Protein phosphatase 1 complex, and Polyadenylation Factor I respectively. White nodes are not assigned in any benchmark.

The above experiments measure the performances based on various metrics and the results show that the different protein complex identification methods rank differently for different metrics. Thus it is difficult to comment on the best performing algorithm. In this section, we evaluate the performances of the methods using an aggregated ranking procedure. We use three topological measures namely density, betweenness centrality and clustering coefficient and a biological relevance measure namely GO based semantic similarity for ranking each complex. Subsequently, a rank aggregation scheme^36^ is used to aggregate the individual ranks. A comparison between the predicted complexes identified by the proposed method and that by the clusterONE is reported in Table 5. The Table 5 shows the modules having top five ranks, their matched benchmark complexes and proportion of proteins involved in some benchmark complexes. As can be seen from the table that predicted complexes of the proposed method have higher proportion of involvement in the benchmark than clusterONE. From Table 5 it can be noticed that the complexes: COMPASS, histone H3 methyltransferase protein complex, Protein phosphatase 1 complex and Polyadenylation Factor I are detected by the top rank predicted complex-2. Figure 9 further shows the predicted complex and the proteins involved in three different benchmark complexes.

**Table 5:** Table shows the comparison of top rank five predicted complexes of proposed and clusterONE method. Second column represents benchmark complexes detected by the methods.

Top ranked predicted complex		Matched complex		% of involvement in benchmark complex	
proposed	clusterONE	proposed	clusterONE	proposed	clusterONE
module-2	module-90	COMPASS, histone H3 methyltransferase protein complex (66.67%), Protein phosphatase 1 complex (53.33%), Polyadenylation Factor I (48.15%)	Nup84 sub-complex(45%), Complex 228 (18%)	72.97%	59.79%
module-84	module-40	Heteromeric p24 complex 1 (25%), Complex 339 (19%)	Actin capping complex (25%)	66.67%	52.38%
	7	complex-302 (35%)	Complex 263 (38%)	20.89%	18.18%
module-78	module-10	TRAPPII complex (25%), Kel1/Lte1 complex (18.33)%	Complex 263 (25%), Complex 215(20%)	40%	38.83%
module-112	module-235	Complex 482 (35%), Complex 436(21%)	Kap104/Hrp1 complex (50%), COMPASS, histone H3 methyltransferase protein complex (25%)	40%	52.4%
module-109	module-02	Heteromeric p24 complex 1 (83.35%), Sin3 Histone deacetylase complex (23%)	Complex 457 (40%) Complex 444 (33.33%)	62.22%	58.93%

Third column represents proportion of proteins that are involved in some benchmark complexes.

## Discussion

Identifying protein complexes from protein-protein interaction (PPI) data is an important problem in computational biology. The existing literature resulted from a cross-disciplinary research, proposes several methods based on global objective maximization, network motif identification, local search mechanisms etc. In this paper we propose a sequential non-cooperative game based model for protein complex detection from PPI network. We model protein complex identification as a non-cooperative sequential partial dense vertex cover game. The Nash equilibrium of the game corresponds to a minimum partial dense vertex cover of the given network.We carry out a thorough experiment for validation of topological and biological relevance of the identified complexes. The data sets used in the experiment are synthetic benchmarks and real life yeast PPI data. The experiments on synthetic benchmark network and null-models proves the accuracy of the proposed method. We further test robustness of the proposed model on perturbed synthetic benchmarks where edges are rewired with certain probability. The results prove that the developed model accurately identifies the network module even when the rewiring probability is moderately high. The tests on real PPI networks also shows promising results. The validation through connectivity density proves that the identified modules on the real data are dense indeed and good candidates for protein complexes. The partitions are further validated using matching score, sensitivity, positive predictive value and accuracy metrics against experimentally annotated data. Although the proposed method does not outperformed all other state-of-the-art with respect to the performance metrics, but it achieves a consistently good score for all of the datasets. It is noticeable that, although RNSC and MCL achieve higher accuracy than the proposed method in MIPS and KROGAN data, they are not performing well while comparing with respect to the functional similarity score. The experiment shows that the proposed method attains high functional similarity score for most of the identified modules. Additionally, from the experiment we find a common drawback with the existing approaches is that they find a few well-matched clusters and a high number of poorly matched clusters. The proposed approach overcome this issue and finds the clusters whose matching score is far less skewed compared to the existing approaches. Finally we measure the performance using an aggregated ranking approach which proves the superiority of the proposed method over the base-line methods. It is noticeable that the proposed approach is also capable to identify sparse, low dense protein complexes. Setting the input parameters *α* and *β* to a low value, the proposed method results low dense clusters which may be treated as possible candidates for sparse, low dense protein complexes.

Applying game theory for sub-cellular events like protein-protein interactions is a rather new approach of research. For simplicity, the proposed method is designed to detect the disjoint complexes. However, with the use of appropriate data structure and by removing the restriction that if a protein joins a coalition, then the protein is removed from the game, the proposed method can be extended to detect the overlapping complexes. In future we would like to study the weighted PPI networks for the analysis of protein complexes and functional modules.

## References

[1] Srihari S, Leong HW (2013). A survey of computational methods for protein complex prediction from protein interaction networks. Journal of bioinformatics and computational biology.

[2] Nepusz T, Yu H, Paccanaro A (2012). Detecting overlapping protein complexes from protein-protein interaction networks. Nature Methods.

[3] Mirny L, Spirin V (2003). Protein complexes and functional modules in molecular networks. Proc. Natl Acad. Sci.

[4] Hartwell LH, Hopfield JJ, Leibler S, Murray AW (1999). From molecular to modular cell biology. Nature.

[5] Zhang B, Park B-H, Karpinets T, Samatova NF (2008). From pull-down data to protein interaction networks and complexes with biological relevance. Bioinformatics.

[6] Srihari S, Yong CH, Patil A, Wong L (2015). Methods for protein complex prediction and their contributions towards understanding the organisation, function and dynamics of complexes. FEBS letters.

[7] Pereira-Leal J, Enright A, Ouzounis C (2004). Detection of functional modules from protein interaction networks. Proteins.

[8] Altaf-Ul-Amin M, Shinbo Y, Mihara K, Kurokawa K, Kanaya S (2006). Development and implementation of an algorithm for detection of protein complexes in large interaction networks. BMC Bioinformatics.

[9] Brohee S, van Helden J (2006). Evaluation of clustering algorithms for protein-protein interaction networks. BMC Bioinformatics.

[10] Girvan M, Newman M (2002). Community structure in social and biological networks. Proceedings of the National Academy of Sciences.

[11] Bader G, Hogue C (2003). An automated method for finding molecular complexes in large protein interaction networks. BMC Bioinformatics.

[12] King AD, Przulj N, Jurisica I (2004). Protein complex prediction via cost-based clustering. Bioinformatics.

[13] Frey BJ, Dueck D (2007). Clustering by passing messages between data points. Science.

[14] Mukhopadhyay A, Ray S, De M (2012). Detecting protein complexes in a ppi network: a gene ontology based multi-objective evolutionary approach. Mol Biosyst..

[15] Bandyopadhyay, S., Ray, S., Mukhopadhyay, A. & Maulik, U. A multiobjective approach for identifying protein complexes and studying their association in multiple disorders. *Algorithms for Molecular Biology* **10**, doi:10.1186/s13015–015–0056–2 (2015). 10.1186/s13015-015-0056-2 PMC4529733 26257820

[16] Widita CK, Maruyama O (2013). Ppsampler2: Predicting protein complexes more accurately and efficiently by sampling. BMC systems biology.

[17] Zaki, N., Efimov, D. & Berengueres, J. Protein complex detection using interaction reliability assessment and weighted clustering coefficient. *BMC bioinformatics* **14**, doi:10.1186/1471–2105–14–163 (2013). 10.1186/1471-2105-14-163 PMC3680028 23688127

[18] Tatsuke D, Maruyama O (2013). Sampling strategy for protein complex prediction using cluster size frequency. Gene.

[19] Bohl K (2014). Evolutionary game theory: molecules as players. Mol Biosyst..

[20] Dongen, V. *Graph clustering by flow simulation*. Ph.D. thesis, University of Utrecht (2000).

[21] Newman M, Girvan M (2004). Finding and evaluating community structure in networks. Phys. Rev. E.

[22] Gavin A (2002). Functional organisation of the yeast proteome by systematic analysis of protein complexes. Nature.

[23] Krogan N, Cagney G, H. Y. (2006). Global landscape of protein complexes in the yeast saccharomyces cerevisiae. Nature.

[24] Salwinski, L. *et al*. The database of interacting proteins: 2004 update. *Nucleic Acids Res* supl 1, 449–451 (2004). 10.1093/nar/gkh086 PMC308820 14681454

[25] Guldener U (2005). Cygd: the comprehensive yeast genome database. Nucleic Acids Res.

[26] Ana, L. & Jain, A. Robust data clustering. In *Proceedings. IEEE Computer Society Conference on Computer Vision and Pattern Recognition*, vol. 2, II–128–II–133 vol.2 (2003).

[27] Danon L, Diaz-Guilera A, Duch J, Arenas A (2005). Comparing community structure identification. Journal of Statistical Mechanics: Theory and Experiment.

[28] Erdös P, Rényi A (1961). On the strength of connectedness of a random graph. Acta Mathematica Hungarica.

[29] Chen J, Yuan B (2006). Detecting functional modules in the yeast protein-protein interaction network. Bioinformatics.

[30] Brohee, S. & Helden, J. Evaluation of clustering algorithms for protein-protein interaction networks. *BMC Bioinformatics* (2006). 10.1186/1471-2105-7-488 PMC1637120 17087821

[31] Kiemer L, Costa S, Ueffing M, Cesareni G (2007). Wi-phi: A weighted yeast interactome enriched for direct physical interactions. Proteomics.

[32] Ray S, De M, Mukhopadhyay A (2012). A multiobjective go based approach to protein complex detection. Procedia Technology.

[33] Hossain SMM, Mahboob Z, Chowdhury R, Sohel A, Ray S (2016). Protein complex detection in ppi network by identifying mutually exclusive protein-protein interactions. Procedia Computer Science.

[34] Ray, S., Bandyopadhyay, S., Mukhopadhyay, A. & Maulik, U. Incorporating fuzzy semantic similarity measure in detecting human protein complexes in ppi network: A multiobjective approach. In *Fuzzy Systems (FUZZ), 2013 IEEE International Conference on*, 1–8 (IEEE, 2013).

[35] Schlicker A, Domingues F, Rahnenfuhrer J, Lengauer T (2006). A new measure for functional similarity of gene products based on gene ontology. BMC bioinformatics.

[36] Pihur V, Datta S, Datta S (2007). Weighted rank aggregation of cluster validation measures: a monte carlo cross-entropy approach. Bioinformatics.

